# Rabies experts on demand: A cross‐sectional study describing the use of a rabies telehealth service

**DOI:** 10.1002/puh2.109

**Published:** 2023-08-01

**Authors:** Sarah E. Baker, Yasmeen B. Ross, James A. Ellison, Benjamin P. Monroe, Lillian A. Orciari, Brett W. Petersen, Agam K. Rao, Ryan M. Wallace

**Affiliations:** ^1^ Poxvirus and Rabies Branch Division of High‐Consequence Pathogens and Pathology Centers for Disease Control and Prevention Atlanta Georgia USA; ^2^ Oak Ridge Institute for Science and Education Centers for Disease Control and Prevention Participation Program Atlanta Georgia USA

**Keywords:** digital health, prevention and control, public health, rabies, telehealth service, telemedicine, zoonotic disease

## Abstract

**Background:**

Rabies expert on demand (REOD) telehealth service is provided by the U.S. Centers for Disease Control and Prevention (CDC) to assist public health practitioners, health providers, and the public to interpret national and international rabies prevention guidelines. REOD is staffed by subject matter experts of the CDC Poxvirus and Rabies Branch to assess each unique situation and provide evidence‐based guidance to stakeholders. This study aims to describe the utilization of a rabies telehealth system and provide insight into common consultations.

**Methods:**

A cross‐sectional study of the nature of inquiries to REOD was done using the data collected from September 1, 2017 to September 30, 2021. An inquiry tracking form and Microsoft Access database were developed to document all inquiries received. Inquired ones were summarized to determine the frequency of inquiries by month, category, and location.

**Results:**

Over a 49‐month period, REOD received 5228 inquiries. Peak inquiries (*n* = 108) occurred during August 2019. The most frequent inquiries received pertained to risk assessment and management of rabies exposures (*n* = 1109), requests for testing assistance (*n* = 912), consultation for suspected human rabies (*n* = 746), rabies exposures and post‐bite treatment occurring internationally (*n* = 310), and consultation for deviations in the recommended pre‐ and postexposure prophylaxis regimen (*n* = 300).

**Conclusion:**

REOD is a global resource for consultation related to managing rabies exposures, diagnostic issues, and rabies control strategies. REOD is a regularly utilized CDC service, as the demand for up‐to‐date rabies guidance remains high. REOD fulfills a critical role for the interpretation and consultation on rabies prevention guidelines to stakeholder.

## Graphical abstract

The role of REOD is to help patients, providers, and health departments understand the Bio‐Geo‐Behavioral risk assessment processes, so that appropriate and economical health decisions can be made. Most inquiries are received between June and August with questions about rabies exposures being the most frequently asked throughout the year.

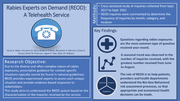

## INTRODUCTION

Rabies is a preventable disease most often transmitted through the bite of a rabid animal and causes an estimated 59,000 human deaths each year globally, primarily from a variant of rabies virus that is maintained in domestic dog populations [1]. In the United States (US), approximately 100,000 animals are tested for rabies annually, and 60,000 people receive rabies postexposure prophylaxis (PEP). Despite the availability of consultations from public health authorities and the widely accessible PEP, typically 1–3 human rabies deaths occur each year in the US [1]. Zoonotic diseases, like rabies, pose a severe threat to the health of humans and their pets. Globally, zoonotic diseases are responsible for over 2.7 million human deaths a year [2]. The link between the health of people and pets is recognized by a One Health approach that acknowledges that the interactions among human, animals, and the environment are critical drivers of zoonoses [3]. Rabies control in the US follows the One Health approach by promoting routine rabies vaccination for domestic pets, educating the public of the potential dangers of interacting with wildlife, ensuring appropriate administration of PEP, and managing rabies in certain wildlife populations [4].

In the US, the dog‐maintained rabies virus variant (RVV) was eliminated in 2007. Since then, about 90% of reported cases of rabies in animals occur in wildlife, and rabies virus reservoir species have been identified in all US states except Hawaii. The National Rabies Surveillance System (NRSS) collects laboratory and epidemiological data from 54 US public health and animal health jurisdictions. The most frequently reported rabid animals submitted in 2019 were raccoons (32.9%), bats (29.6%), skunks (19.5%), and foxes (7.7%). Domestic animals made up 8.2% of all animals confirmed rabid [1].

The Centers for Disease Control and Prevention (CDC) is the leading federal public health agency in the US, and the Poxvirus and Rabies Branch (PRB) serves as the National Reference Laboratory and National Surveillance System for rabies. Over 250,000 people visit the CDC Rabies homepage each year to receive information about rabies. CDC Rabies web content includes messaging for the public on rabies virology, epidemiology, and prevention as well as information for specific groups like doctors, laboratorians, and veterinarians. As access to information has become more readily available and individuals become more health conscious, so has the need for accurate and timely dissemination of health information.

Many state and local health departments have the means and capacity for the public to directly speak with public health experts, often through telephone and email, to address their health concerns. Generally, rabies questions from the public and human or animal healthcare providers should reach out their state health departments to address their rabies concerns. These modes of communication are critical for field‐level reporting of events of public health concern, as well as a means to educate the public on common public health risks, such as rabies. Similarly, PRB has operated the rabies experts on demand (REOD) telehealth services since 2005 with the formal data tracking system described in this report beginning in 2017. The Health Resources Services Administration defines *telehealth* as the use of electronic information and telecommunications technologies to support long‐distance clinical health care, patient and professional health‐related education, public health, and health administration. As such, REOD is a regularly utilized service that assists public health practitioners, health providers, and the public to interpret national and international rabies prevention guidelines.

Although resources for rabies as well as guidance documents are readily available through local, state, and federal health agencies, there are no published summaries of rabies telehealth systems. This study aims to describe the operation and use of the REOD telehealth service and provide insight into some of the most common rabies consultations posed by stakeholders.

This study aims to describe the utilization of a rabies telehealth system and provide insight into common consultations.

## METHODS

### Study design and study setting

This is a cross‐sectional study of inquiries (call or email) to the REOD telehealth services collected from September 1, 2017 to September 30, 2021 (49 months). The content of the data was retrieved from the daily tracking form in the REOD database. An Access database (Microsoft Corporation) and form were created to document inquiries.

**FIGURE 1:**
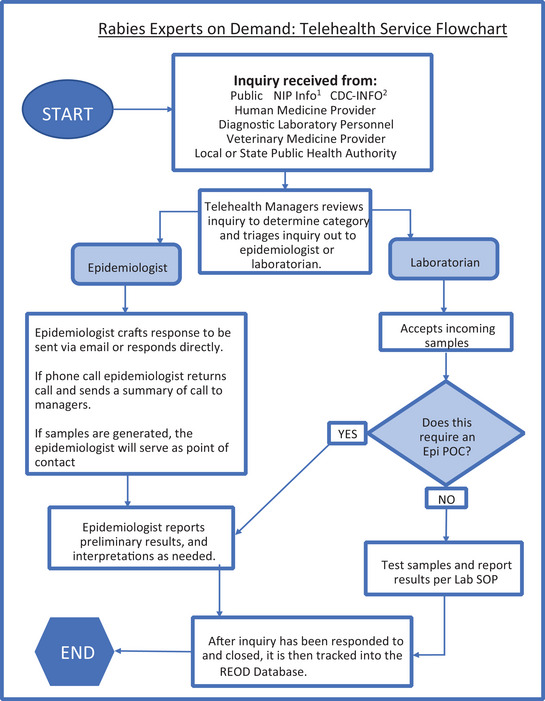
The process of receiving and managing inquiries received to rabies expert on demand (REOD). ^1^NIP‐INFO: The general inquiry line to contact the national immunization and respiratory diseases. ^2^CDC‐INFO: The general inquiry line to the Centers for Disease Control and Prevention.

REOD is staffed by approximately 8–12 PRB staff with expertise in rabies epidemiology, laboratory sciences, veterinary sciences, and human medicine. Inquires received by REOD are submitted by public health officials, clinicians, or general members of the public through one of the following: a monitored email box (rabies@cdc.gov), phone‐line (404‐639‐1050), or CDC's general inquiry lines (e.g., CDC‐INFO or the CDC Emergency Operations Center [EOC]). Direct contact to REOD was communicated to state partners via presentations and word of mouth from rabies epidemiologist. Additionally, REOD was contacted by other internal CDC channels, such as CDC‐INFO, EOC, and NIP‐INFO. Inquiries are reviewed by a REOD manager, categorized by nature of the inquiry (e.g., rule‐out of human rabies and serology request), and triaged to a subject matter expert (SME) best able to address the specific question (Figure 1). The rabies telehealth line is monitored Monday through Friday from 8:00 a.m. to 5:00 p.m., except for national holidays. Inquiries submitted outside of these working hours are routed through the EOC and, depending on the urgency of the inquiry, possibly routed directly to an on‐call rabies expert.

REOD works closely with state and local health authorities to assess each unique situation and, when possible, align recommendations with jurisdictional partners at local and state health departments. National guidelines for rabies prevention include the Advisory Committee on Immunization Practices (ACIP) Human Rabies Prevention Recommendations [5] and the National Association of State Public Health Veterinarians (NASPHV) Compendium for Animal Rabies Prevention and Control [6]. These documents serve as the basis for REOD responses to inquiries. Due to the diverse and often complex nature of rabies exposures, prescriptive guidance for context‐specific situations typically cannot be found in these documents; REOD provides experienced experts to assess each unique situation and provide evidence‐based responses to stakeholders. As a practice, REOD defers all prescriptive treatment decisions to healthcare providers and state health departments; information provided by REOD may help to inform these decisions.

### Study variables and instrument

The form developed characterizes the nature of inquiries and quantify the staffing time required to operate the REOD database (Section S1). For each call or email, the form is filled out with the date the inquiry was received, the name of the SME assigned, inquiry category, and an estimate of the time required for the consultation. Even though it is possible for inquiries to fall into multiple topics, the REOD database only assigns the primary category. Categories are broken into tiers one through three based upon complexity of the topic (Section S1). Tiers are further stratified by the topic of the inquiry. Topics were grouped into nine main categories by the REOD manager based on information provided in the initial inquiry. Individual subcategories were as follows: rabies exposures, animal rabies epidemiology, clinical management of human exposures, deviations in recommendations for pre‐ or PEP, suspected human rabies consults, international rabies, animal serology for pet travel, laboratory testing, and other (Section S2). Although no strict definitions exist for these categories, the REOD manager uses best judgment based on the information provided at initial inquiry to assign a category (Section S3). Only initial inquiries and a final summary of the consultation are tracked in the REOD database; therefore, downstream interim communications stemming from an initial inquiry are not captured in the REOD database or this analysis.

### Data analysis

Descriptive analyses were conducted using Microsoft Excel (Microsoft Corporation). Inquiries were described by the method of contact, category, caller profession, and inquiry source. The call rate per capita for domestic inquiries was calculated based on the human population residing within the states, stratified by the dominant terrestrial reservoir RVV. Six distinct RVVs are geographically associated with the four terrestrial wildlife reservoir species in the US, which includes raccoons (*Procyon lotor*), skunks (family *Mephitidae*), foxes (*Vulpes* spp. and *Urocyon cinereoargenteus*), and the small Indian mongoose (*Herpestes auropunctatus*) exclusively in Puerto Rico. These territories are as described in Figure 1 of [1]

### Ethical considerations

Inquiries to REOD are tracked as part of normal practices to monitor programmatic needs and inform the Rabies Program of potential gaps in guidance. No identifying information is collected; therefore, there is no authority that is required to grant approval for a descriptive analysis such as this.

## RESULTS

**FIGURE 2:**
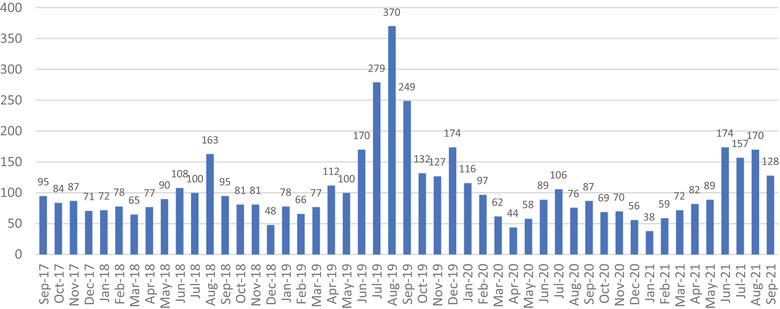
Frequency of inquiries received by month to rabies expert on demand (REOD) between September 2017 and September 2021.

Over a 49‐month period, REOD received a total of 5228 inquiries; 3777 (72%) were received by email and 1451 (28%) by phone. Frequency of calls by month is shown below in Figure 2, peak monthly inquiries (*n* = 370) occurred in August 2019. On average, REOD consulted on rabies exposures 23 times per month (total *n* = 1109), for clinical management of humans 15 times per month (total *n* = 746), and international rabies 6 times per month (total *n* = 310). Laboratorians were contacted on average 19 times per month (*n* = 912) to request assistance interpreting diagnostic results at state or local laboratories.

The characteristics of inquiries received by the REOD telehealth service are described in Table 1. The highest volumes of calls and emails were received from state and local health departments (35%), followed by the public (32%). The primary form of contact is direct contact (69%) to our phone line or rabies inbox via email, followed by CDC‐INFO inquiries (21%). The majority of inquiries were received from US‐based agencies and residents (89%) compared to 11% received from international sources. On average it took 30 min to triage, respond, and track an inquiry. It is estimated that 462 h of staff time (46 h per SME) was spent annually on responding to initial inquiries.

**TABLE 1:** Characteristics of inquiries by rabies expert on demand (REOD) between September 1, 2017 and September 30, 2021 (*n* = 3401).

Characteristics	*n*	%
**Stakeholder**		
Human medicine provider	614	18
Diagnostic laboratory personnel	372	11
Local or state public health authority	1176	35
Public	1096	32
Veterinary medicine provider	316	9
Other	92	3
**Route to REOD**		
CDC‐INFO^b^	717	21
Direct contact—email	1139	33
Direct contact—phone call	1210	36
EOC	113	3
NIP‐INFO^a^	222	7
**Origin**		
Domestic (United States)	3013	89
International	388	11
**Categories of questions**		
Animal rabies epidemiology	288	8
Clinical management of human exposures	746	22
International rabies	310	9
Laboratory testing	912	27
PrEP/PEP deviations	300	9
Animal serology	260	8
Rabies exposures	1109	33
Suspected human rabies	257	8
Other	351	10

Abbreviations: EOC, Emergency Operations Center; PEP, postexposure prophylaxis; PrEP, pre‐exposure prophylaxis.

^a^ NIP‐INFO: The general inquiry line to contact the national immunization and respiratory diseases.

^b^ CDC‐INFO: The general inquiry line to the Centers for Disease Control and Prevention.

The top five most common inquiries came from the following categories: (i) rabies exposures (33%), (ii) laboratory testing (27%), (iii) clinical management of human exposures (22%), (iv) international rabies (9%), and (iv) PEP deviations (9%). Laboratory testing inquiries were further subcategorized by those that resulted in samples being submitted to CDC's National Rabies Reference Laboratory and those that required remote technical consultation. Samples that originate from humans or animal samples in which a human exposure occurred are considered CLIA samples.

The greatest number of calls originated from states enzootic for the raccoon RVV (Table 2). The highest call rate per capita for areas with more than 1 million people were the raccoon territory (2.1. Hawaii, the only state considered rabies free, had the lowest call rate at 0.7).

**TABLE 2:** Call rate per capita for domestic inquiries received between September 1, 2017 and September 30, 2021.

Enzootic rabies virus variant	Inquiries received from region	Human population of the region^a^ with the enzootic rabies virus variant	Average annual rate of inquiries (per million)	95% CI
**Raccoon**	1213	139,833,064	2.1	2.0–2.3
**Skunk‐only**	934	138,590,904	1.7	1.6–1.8
**Skunk/gray fox**	44	7151,502	1.5	1.1–2.0
**Arctic fox (AK)**	25	733,391	8.3	5.5–12.1
**Mongoose**	17	3285,874	1.3	0.8–2.1
**Bat‐only**	261	40,878,082	1.6	1.4–1.8
**Rabies free (HI)**	4	1455,271	0.7	2.1–16.24
**Total**	2934	330,000,000	2.2	

^a^ Bureau, U. C. (n.d.). *STATE PROFILES 2020 Census*: The United States Census Bureau. https://www.census.gov/library/stories/state‐by‐

state.html.

**FIGURE 3:**
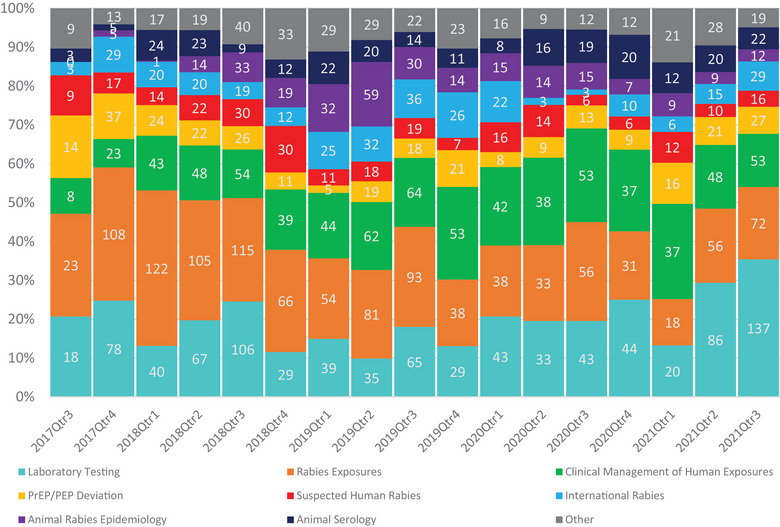
Types of questions received by rabies expert on demand (REOD) by quarter between September 2017 and September 2021.

Based on the peaks shown in Figure 2, we observed a seasonal trend in the number of inquiries received, with the greatest number received during summer months, June through August. Over the 49‐month period, inquiries peaked on average in August with 108 inquiries per month. February had the lowest amount of average inquires with 52 per month. This pattern is apparent in 2018, 2019, and 2021. As shown in Figure 3, the types of inquiries received vary based upon the time of year. Questions regarding rabies exposures are the most common type of question responded to year‐round. Questions surrounding clinical management peaked around quarter three of each year as seen in 2018 (*n* = 54), 2019 (*n* = 64), and 2020 (*n* = 53).

## DISCUSSION

Human rabies deaths are rare in the US, but wildlife rabies and human exposures are abundant. A large number of calls are fielded by REOD, annually; these are inquiries that required rabies expert interpretation and case‐by‐case consultations. The most common inquiries were risk assessments of suspected rabies virus exposures, followed by the acceptance of samples for laboratory testing, and human rabies rule out consultations. In warmer months such as June to August, there is a higher call volume due to more frequent outdoor activity, thus increasing the likelihood of interactions between humans and animals. A notable decline in consultations occurred in 2020, which could presumably be due to COVID‐19, which changed people's travel, leisure, and healthcare seeking behaviors as well as inundated public health systems. Supporting this assumption, the three north American countries (Canada, US, and Mexico) all reported significantly lower rabies testing requests in 2020 and linked this to pandemic strains on public health infrastructure [1]. Maintaining rabies control infrastructure at the local, state, and federal levels is critical for human and animal health, with some estimates suggesting that this infrastructure provided a net savings of $500 million each year by reducing costs associated with treatment for rabies virus exposures and preventing human and livestock deaths [5]. The REOD is one component of this infrastructure and has shown to be a regularly utilized method for addressing a plethora of rabies issues faced by health departments, the public, and international partners. The following discussion provides insight into the process by which REOD addresses some of the most common consultations.

### Rabies exposures

Risk assessments for suspected rabies exposures were the most frequent inquiry received by REOD during the study period. Rabies is an invariably fatal viral disease that must be treated prior to symptom onset to avert death. Bites are the most frequent mode of rabies virus transmission, but non‐bite transmission can occur such as through viral exposure to fresh open wounds or mucous membranes or through organ and tissue transplantation [7]. It is estimated that 55,000 Americans receive PEP each year and although cost varies, a full course of PEP (RIG and vaccine) cost on average $3800 not including the cost of medical treatment or wound care [8]. REOD SMEs utilize a Bio‐Geo‐Behavioral process to evaluate the risk of rabies after a potential exposure.

Biological risk assessment: Only mammals transmit rabies virus, and not all mammals pose equivalent risk of transmission. Certain species are reservoirs for rabies virus and pose a high risk of infection, whereas others have unfavorable host and ecological factors that make them less susceptible to infection and onward transmission. For example, it is hypothesized that the low resting body temperature of opossums makes them a less favorable host for rabies virus, and epidemiologic data supports these findings given the very low rate of rabies in this particular species [9]. PRB maintains a historical record of all reported rabid animals since the 1950s, which provides a robust data repository for assessing the risk of rabies based on animal biology. Understanding these biological and taxonomic differences in risk of infection can help inform the rabies risk assessment process.

Geographical assessment: Rabies risks can also be differentiated based on the geography where the exposure took place. Rabies epidemiology in the US displays a clear species and geographical pattern. Understanding this pattern is important when evaluating the risk posed by a suspected rabies exposure. Raccoons involved in human exposure events in Eastern coastal US states have an 18% chance of having rabies virus infection, whereas raccoons in other parts of the US are rarely infected with rabies. PRB produces a rabies risk map each year to inform the geographical considerations of rabies risk assessments [1].

Behavioral assessment: Rabies in affected animals typically manifests with notable aberrant behaviors. The degree of aberrant behavior an animal may display can vary from normal acting to extreme behavioral and physical abnormalities. Therefore, behavior alone should not be used to rule out the need for PEP. However, understanding the nature of the exposure (provoked vs. unprovoked) and clinical signs observed in the offending animal is an important part of the risk assessment process.

REOD defers all bite treatment decisions to medical providers and local/state health departments. The role of REOD is to help patients, providers, and health departments understand this Bio‐Geo‐Behavioral risk assessment process, so that appropriate and economical health decisions can be made, and the bite victim does not incur undue financial burden when there is no risk of rabies.

### Laboratory inquiries

PRB serves as the National Rabies Reference Laboratory. As such, PRB acts as a central resource for diagnostic laboratory personnel and health departments for issues such as confirmatory testing, antigenic and genetic virus characterization, and atypical testing procedures (e.g., immunohistochemistry test on formalin‐fixed tissues). There are several common reasons the National Rabies Reference Laboratory is requested for consultation. First, timely and accurate diagnosis of animal samples is essential to provide appropriate treatment recommendations. Reference confirmatory diagnostics involves assistance to rabies laboratories by repeat testing of the sample(s) at CDC with multiple tests or reagents to rule‐out rabies (i.e., DFA and real‐time PCR), and often explaining or advising on the interpretation of test results with either specific or nonspecific outcomes. It may involve explanation of reagent optimization procedures or assistance with obtaining test materials or reliable alternatives when in short supply, and all these activities impact the final test results.

Although it is critical to accurately diagnose animals with rabies, reporting of negative test results prevents the unnecessary administration of PEP and the associated financial burden; the PEP series can cost over $5000 per person [10]. Second, the epidemiology of rabies provides the basis of rabies management operations and public health recommendations. The characterization of RVVs is often performed to detect epidemiologic shifts and ensure that appropriate public risk communication is enacted. CDC has defined baseline criteria for when samples should undergo additional testing for RVV characterization [11, 12]. When rabies laboratories lack the financial means or technical capacity for viral characterization, the National Rabies Reference Laboratory may be utilized.

### Human rabies rule out consultations

Since 1944, rabies in humans has been a nationally notifiable disease, the CDC is to be notified within 24 h of a confirmed human rabies case [13]. As with many rare diseases, successful control efforts have resulted in a healthcare system that rarely cares for patients with this disease. As the National Rabies Reference Laboratory and NRSS, REOD routinely advises on clinical and laboratory diagnosis of rabies, exposure investigations including contact tracing and risk assessments, and transmission prevention recommendations.

Rabies pathogenesis is well described in the context of clinical timeline and health outcome (death). Therefore, human rabies consultations are based on key criteria, including the history of a potential exposure, the current health status of the patient, clinical course of illness, a diagnosis of encephalitis, and other disease testing to rule out more common causes of encephalitis. Given the rare nature of human rabies in the US, other plausible causes should usually be ruled out prior to antemortem rabies testing. The presentation of human rabies can vary greatly, and REOD is available to assist states with clinical consultations, interpretations of recommendations, and ante‐ or postmortem diagnostic testing.

### PEP outside the United States

Exposure to rabid dogs while traveling internationally is one of the leading causes of rabies death in the US [1]. It is highly recommended when traveling to an international country to research whether rabies is present in the dogs and wildlife [10]. If a US citizen has had an exposure to a rabid animal while abroad, the US Embassy located in that country may be available to help locate medical services such as a healthcare provider and state and local public health officials to treat and provide PEP [14]. The Embassy can also assist if continued care is needed. REOD can be notified to alert the State Health Department in the home state of the patient to provide treatment after the return to the US.

Rabies vaccines administered in other countries may not be U.S. Food and Drug Administration (FDA) approved [15]; therefore, REOD can provide a consultation to human health providers on a case‐by‐case basis to determine if additional vaccines or verifications by titer are necessary of the vaccines for both pre‐exposure and PEP. In most cases, if there is a concern about the validity of a vaccine received outside of the US, or if multiple vaccine products are administered, REOD advises to verify an adequate antibody response through a rabies virus neutralization test.

### PEP deviations

When a rabies exposure cannot be reasonably ruled out through laboratory testing or a risk assessment, persons are typically recommended to initiate PEP. For individuals who have not completed rabies pre‐exposure prophylaxis, the series is comprised of a multi‐injection regimen of human rabies immune globulin (weight‐based dosing) and vaccine on the first healthcare visit, followed by three additional vaccine injections on days 3, 7, and 14. Immunocompromised individuals are recommended to receive a fifth vaccine dose on day 28 and have their immune response verified through serologic testing for rabies neutralizing antibodies. Given the multi‐visit nature of rabies PEP, deviations in this schedule are common and occur for diverse reasons ranging from inability to schedule weekend vaccinations to forgetfulness. The ACIP recognizes that minor delays in the timing of doses do not impact the rapidity or robustness of the immune response. However, longer delays of weeks or months are not well studied and could lead to delayed or reduced antibody response. Either of these scenarios could be fatal for those exposed to rabies virus.

In an effort to provide guidance on this common inquiry, REOD developed an excel‐based tool that allows users to input the rabies PEP schedule that the patient experienced or a schedule that is proposed (Section S4). The tool acts as a guide to help calculate the days between vaccine doses and determines if a significant deviation from the ACIP‐recommended schedule may have occurred. For large deviations, persons are typically recommended to administer the dose that was delayed and resume the vaccination schedule with original inter‐dose spacing and verify that an adequate immune response was achieved through a rabies virus neutralizing antibody titer test. In rare cases, it may be necessary to restart the entire series.

Common deviations include the injection of rabies vaccine and rabies immunoglobulin in the same anatomical site. Human rabies immune globulin is prepared from human plasma from donors hyper‐immunized with rabies vaccine. The same vaccines used to create rabies immune globulin are also provided to persons with rabies exposures; thus, these antibodies have high affinity for binding vaccine antigen. When they are injected too close, antibody–vaccine complexes neutralize both products, rendering the patient without any meaningful protection from infection [16]. Providers must be cautious about overprescribing rabies immunoglobulin, as studies have shown that administering greater than twice the recommended dose can reduce the immune response to vaccination [17]. When a significant deviation in the PEP schedule has occurred, consultation with a healthcare professional and health department should be pursued to ensure that appropriate recommendations are implemented.

## CONCLUSION

Official and public usage of the telehealth system has grown since its inception in 2005. Documenting inquiries to the telehealth system provided evidence for CDC to support a dedicated staff member to ensuring that inquiries were responded to in a timely manner. This staff member led to the ability to further structure the telehealth system, including training materials, standard responses, and better monitoring. As with all public health efforts, monitoring and evaluation is a critical activity to ensure that resources are used effectively.

Rabies epidemiology is diverse, and animal bites are common. Therefore, ready access to experts for consultations is an important component of the one health approach to rabies control. Fortunately, as a result of robust public health infrastructure, human rabies deaths are very rare in the US, and many healthcare providers and health departments will never encounter a case of human rabies. When these rare events do occur, REOD SMEs can supplement local capacity and assist with diagnostic laboratory testing and case investigations. REOD is a global resource for consultation related to managing rabies exposures, diagnostic issues, and rabies control strategies. REOD is a regularly utilized CDC service, as the demand for up‐to‐date rabies guidance remains high. REOD fulfills a critical role for the interpretation and consultation on rabies prevention guidelines to stakeholder.

## AUTHOR CONTRIBUTIONS

*Conceptualization; data curation; formal analysis; investigation; methodology; project administration; resources; validation; visualization; writing—original draft; writing—review and editing*: Sarah E. Baker. *Data curation; formal analysis; investigation; methodology; project administration; resources; validation; visualization; writing—original draft; writing—review and editing*: Yasmeen B. Ross. *Data curation; investigation; writing—review and editing*: James A. Ellison, Benjamin P. Monroe, Lillian A. Orciari, Brett W. Petersen, Agam K. Rao. *Conceptualization; data curation; formal analysis; investigation; methodology; project administration; resources; supervision; validation; visualization; writing—original draft; writing—review and editing*: Ryan M. Wallace.

## CONFLICT OF INTEREST STATEMENT

The authors declare that there are no conflicts of interest that could be perceived as prejudicing the impartially of the research reported.

## FUNDING INFORMATION

There are no funders to report for this submission.

## DISCLAIMER

This project was supported in part by an appointment to the Research Participation Program at the Centers for Disease Control and Prevention administered by the Oak Ridge Institute for Science and Education through an interagency agreement between the U.S. Department of Energy and the Centers for Disease Control and Prevention. The findings and conclusions in this report are those of the author(s) and do not necessarily represent the views of the Centers for Disease Control and Prevention.

## Supporting information

Supporting Information

Supporting Information

## Data Availability

The data is available upon request.
