# Supplementary material for: Rabies experts on demand: A cross-sectional study describing the use of a rabies telehealth service
Source: Public Health Chall. Author manuscript; Available in PMC 2024 Jan 8. (PMC10772938; doi:10.1002/puh2.109)
Supplement: S1-4. Daily Tracking Form, General Categories of Inquiries, Definitions of Categories [file NIHMS1926574-supplement-S1-4__Daily_Tracking_Form__General_Categories_of_Inquiries__Definitions_of_Categories.docx]

# Supplement File:

## S1. Daily Tracking Form used in Microsoft Access (REOD) Database to collect daily inquiries received by REOD


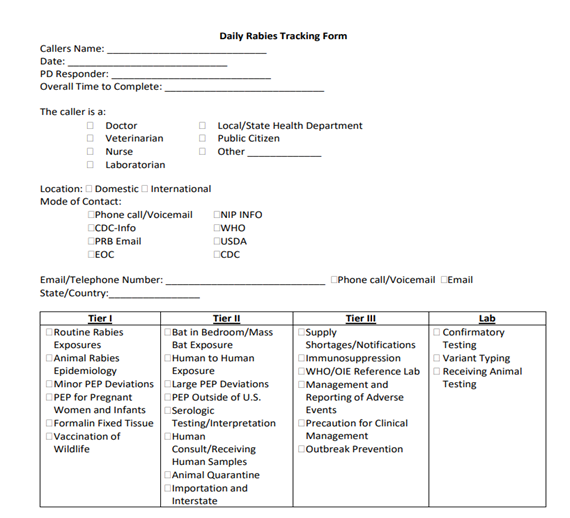


## S2**.** 9 General Categories of Inquiries

| **Laboratory Testing** | **Total** |
| --- | --- |
| Confirmatory Testing | 353 |
| Variant Typing | 94 |
| Receiving Animal Testing | 433 |
| Formalin Fixed Tissues | 32 |
|  | **912** |
| **Rabies Exposures** |  |
| Bat in Bedroom/Mass Bat | 257 |
| Routine Rabies Exposure (non-bat) | 852 |
|  | **1109** |
| **Clinical Management of Human Exposures** |  |
| Immunosuppression | 55 |
| Serologic Testing/Interpretation | 379 |
| Human-to-Human Exposure | 10 |
| Precautions for Clinical Management | 130 |
| Outbreak Prevention | 2 |
| Management and Reporting of Adverse | 62 |
| PEP for Pregnant Women and Infants | 82 |
| Supply Shortage | 26 |
|  | **746** |
| **PrEP/PEP Deviation** |  |
| Large PrEP/PEP Deviation | 66 |
| Minor PrEP/PEP Deviations | 234 |
|  | **300** |
| **Suspected Human Rabies** |  |
| Human Consult/Receiving Human Samples | 257 |
|  | **257** |
| **International Rabies** |  |
| World Health Organization/World Organization for Animal Health | 29 |
| PEP Outside of US | 281 |
|  | **310** |
| **Animal Rabies Epidemiology** |  |
| Animal Rabies Epidemiology | 228 |
| Vaccination of Wildlife | 8 |
| Animal Quarantine | 52 |
|  | **288** |
| **Animal Serology** |  |
| Importation and Interstate | 260 |
|  | **260** |

## S3. Definitions of Categories

| **Supp. 3 – Definitions of Categories**   \| **Animal Rabies Epidemiology** \| Inquiries that require a consultation from a rabies SME to a veterinary medicine provider regarding animal rabies epidemiology, animal quarantine and vaccination of wildlife. \| \| --- \| --- \| \| **Animal Serology** \| Laboratory testing for the importation of animals. \| \| **Clinical Management of Human Exposures** \| Inquiries that require attention from a senior level rabies SME to provide advanced consultation to a human medicine provider or local/state public health authority for clinical management of immunocompromised patients, outbreak prevention, and the reporting of adverse reactions. \| \| **International Rabies** \| Inquiries of rabies exposures that have taken place outside of the U.S. and require guidance from a rabies SME. \| \| **Laboratory Testing** \| Inquiries that require attention from a rabies diagnostic laboratory personnel regarding general lab inquires, confirmatory testing or variant typing for animal testing. \| \| **PrEP/PEP Deviations** \| Consultation to a human medicine provider or local/state public health authority for a deviation on the rabies pre or post exposure prophylaxis series. \| \| **Rabies Exposures** \| Inquiries that require a consultation from a rabies SME to determine the risk level after a potential rabies exposure and to provide the recommended guidance for treatment. \| \| **Suspected Human Rabies** \| Consultation to a human medicine provider or local/state public health authority for a suspect human rabies case. \| \| **Other** \| Inquiries that do not fall within the scope of the other categories such as website updates, data request, and media inquiries. \| |
| --- | --- | --- | --- | --- | --- | --- | --- | --- | --- | --- | --- | --- | --- | --- | --- | --- | --- | --- |

## S4. Deviation Calculator

See attachment
